# Congenital Hairy Polyp Associated with Cleft Palate—A Rare Entity

**DOI:** 10.5005/jp-journals-10005-1042

**Published:** 2009-04-26

**Authors:** Gokul S, Veda Hegde, Kaveri Hallikeri

**Affiliations:** 1 Postgraduate, Department of Oral and Maxillofacial Pathology, SDM College of Dental Sciences and Hospital, Sattur, Dharwad Karnataka, India, e-mail: drgokuls@gmail.com; 2 Assistant Professor, Department of Oral and Maxillofacial Pathology, SDM College of Dental Sciences and Hospital, Sattur Dharwad, Karnataka, India, e-mail: hegdeveda6@rediffmail.com; 3 Associate Professor, Department of Oral and Maxillofacial Pathology, SDM College of Dental Sciences and Hospital, Sattur Dharwad, Karnataka, India, e-mail: drcauveri2005@yahoo.co.in

**Keywords:** Hairy polyp, cleft palate, dermoid, lipoma, teratoma.

## Abstract

Hairy polyps are common congenital benign lesions of oroand nasopharynx containing elements of both ectodermal and mesodermal origin. However, their occurrence in palate is quite rare. Here we present a case of hairy polyp associated with palatal cleft in an eight months old female infant. We discuss the clinicopathological features, etiology, proposed theories related to its formation and its significance.

## INTRODUCTION

Congenital tumors of oral cavity are rare and among those that occur, hairy polyp is the most common, affecting oroand nasopharynx. The total number of documented cases is 135.^1^ They were first classified by Arnold in 1870.^2^ They are benign lesions occurring predominantly in females usually present at or shortly after birth and are rare in elderly age groups. The involvement of other sites in oral cavity is quite uncommon. The constituents of hairy polyp are derived exclusively from the ectoderm and mesoderm and consequently these lesions are classified as dermoid.^1^ We report a rare presentation of hairy polyp associated with cleft palate in a female infant.

## CASE REPORT

**Fig. 1.**
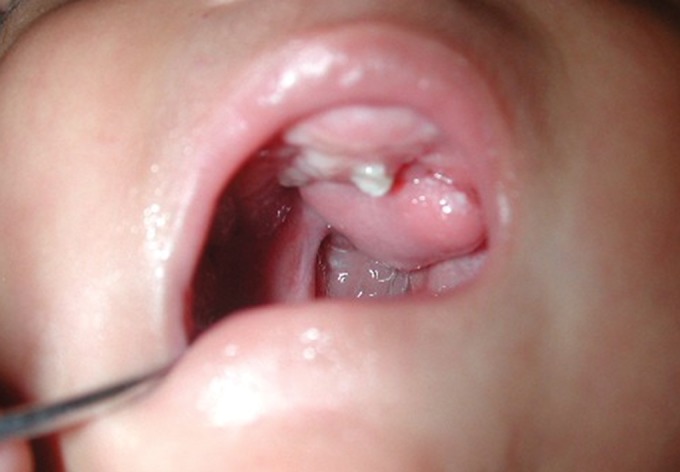
Soft tissue growth on hard palate with cleft of soft palate. Protrusion of premaxilla seen and a single incisor present

An 8 months old female infant was admitted to craniofacial unit of our institution with a growth present on the hard palate. The growth was present since birth and gradually increased in size. Examination of the oral cavity revealed a soft tissue growth measuring about 2 × 2 cm in size on the hard palate associated with cleft of the soft palate. The growth was pedunculated, overlying mucosa was normal with pigmentation seen around the base of the lesion (Fig. 1). There was history of nasal regurgitation and difficulty in feeding. As per family history the marriage was non-consanguineous with normal childbirth. The general condition of the patient was normal. The lesion was provisionally diagnosed as lipoma/ dermoid cyst.

**Fig. 2.**
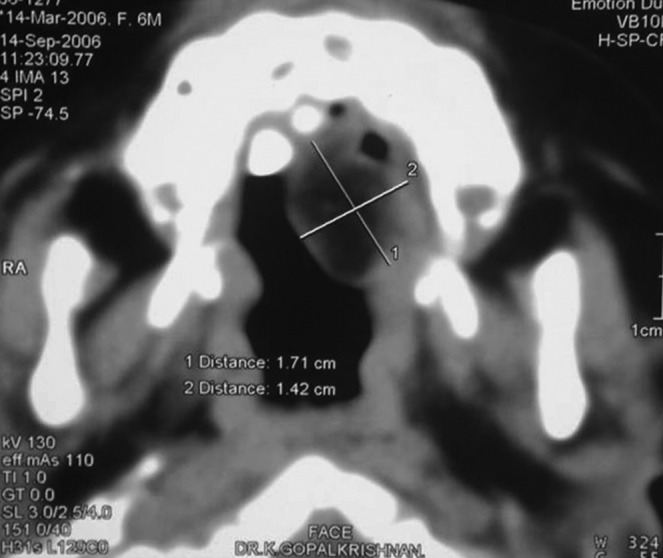
Well-defined solitary hypodense lesion attached to the hard palate

CT-scan revealed the presence of a well-defined solitary, round to oval lesion attached to the hard palate with imaging characteristics of hypodensity compared to that of adjacent bone and muscle (Fig. 2).

The lesion was surgically removed under general anesthesia along with the closure of the palatal defect using the Langenback’s technique.

**Fig. 3.**
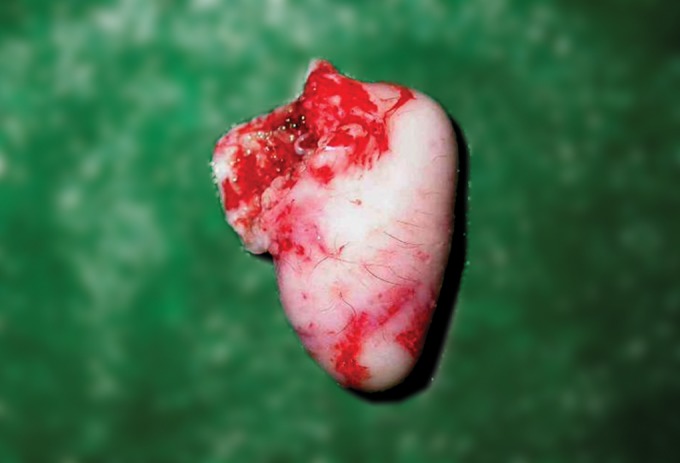
Grayish-white specimen with hair growth on its surface

Gross examination of the excised specimen measured 3 × 2 cm, grayish white in color with smooth surface. The surface of the lesion showed hair growth. The lesion exhibited rubbery consistency (Fig. 3).

**Fig. 4.**
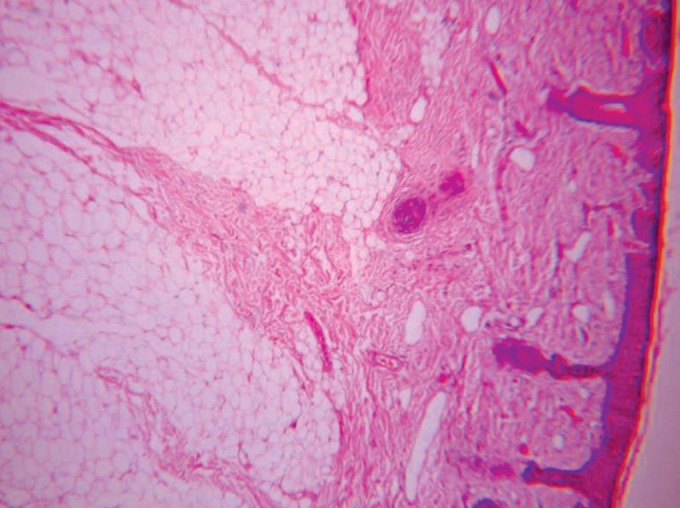
Photomicrograph (4X): Parakeratinized stratified squamous epithelium with immature hair follicles

**Fig. 5.**
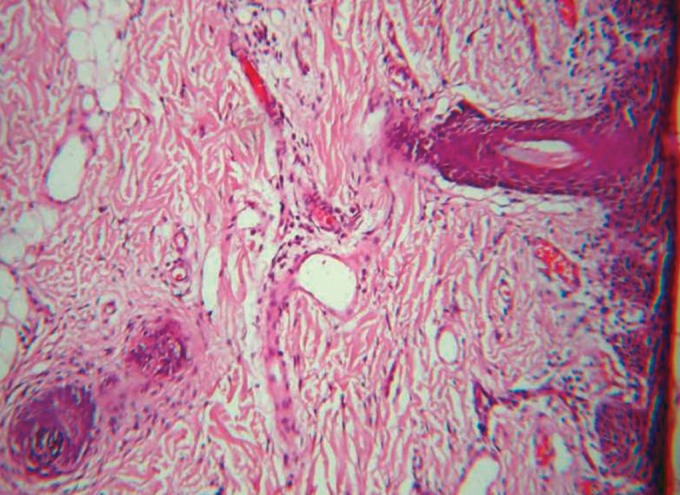
Photomicrograph (10X): Shows immature hair follicles, sweat glands and fat cells in fibrous connective tissue stroma

Microscopic examination revealed epithelium of parakeratinized stratified squamous type (Fig. 4) with immature hair follicles, sebaceous and sweat glands in fibrous connective tissue stroma (Fig. 5). Vacuolated cells are seen in the follicle and lobules of adipose tissue are present.

Based on the histopathological findings, a final diagnosis of hairy polyp was given. The postoperative course was uneventful and the patient was discharged on 5th postoperative day. On follow-up the patient is free of disease, with normal feeding and without evidence of recurrence after 6 months.

## DISCUSSION

Hairy polyps are unusual but well-recognized entities of oro- and nasopharynx.^2^ They were rarely associated with any particular congenital syndrome nor there any predispositions or relationships.^1^ But they were sometimes associated with certain congenital abnormalities such as cleft palate, agenesis of the uvula, external auricle, ankyloglossia, facial hemihypertrophy and left carotid artery atresia.^2^,^3^

Arnold first classified neoplasms arising from nasopharynx as dermoid, teratoid, teratomas, epignathi.^1^There is considerable confusion in the literature regarding the classification of hairy polyp. They have been described as teratomas, hamartomas, dermoids and choriostomas.^3^ Some authors feel a distinction should be made between teratomas and hairy polyp in that the former are neoplastic, show progressive growth and have potential to metastasize.^3^

Hairy polyps are typically pear or sausage shaped lesions that may be sessile but more often are pedunculated. Macroscopically, the size may vary from 0.5 cm to 6 cm and are usually gray or pink in color. Radiological studies are crucial to help delineate the extent and origin of the lesion. Bony anomalies and dehiscence are best identified by CT scanning.^1^ On microscopic examination, they are covered by stratified squamous epithelium and contain skin appendages like hair, sebaceous glands and sweat glands. The fibro fatty stroma may contain striated muscle, cartilage, bone, nerves, lymph follicles and salivary gland tissue. Both serous and mucous glands may be present.

The clinical, gross and histopathological findings in the present case were similar to the description mentioned above. Also CT findings rule out the bony involvement of the lesion.

Various theories have been proposed regarding the hairy polyp formation.^1^

- Disturbed development during the fusion of epiblast of the stomatodeum with anterior foregut
- Failure of nasopharyngeal membrane to regress during the seventh week of gestation
- Misdirected pluripotential tissue
- Misdirected first pharyngeal apparatus in germ cell rests.^4^

As evident from the histopathological features of this case, the constituents of the lesion were derived exclusively from the ectoderm and mesoderm and hence the tumor can be correctly designated as hairy polyp.

## CONCLUSION

Although hairy polyp occurring in nasopharynx has been reported previously, such lesions occurring in the palate is quite rare. Our case is unique in that it is present in association with cleft palate. This is the first such case in more than 1000 cleft palate cases reported in our institution. Also very few cases have been reported in the literature.

- The presence of cleft palate along with hairy polyp may play significant role in the etiopathogenesis of the cleft palate.^5^
- The cleft palate seen in our case might have been the result of failure of closure of palatal shelf caused by the presence of the mass.^6^

It is hence necessary to distinguish hairy polyp as a separate entity because of its clinical significance and microscopic features and they should be considered as developmental malformations.
